# Retraction of: MiR-519d targets HER3 and can be used as a potential serum biomarker for non-small cell lung cancer

**DOI:** 10.18632/aging.206265

**Published:** 2025-05-31

**Authors:** Aili Wang, Hongxia Zhang, Jinxiang Wang, Shuming Zhang, Zhenyang Xu

**Affiliations:** 1 Department of Respiratory and Critical Care Medicine, Beijing Luhe Hospital, Capital Medical University, Beijing 101100, P.R. China

**Keywords:** serum biomarker, miR-519d, non-small cell lung cancer, HER3

**This article has been retracted:** Aging has completed its investigation of this paper. Following communication with the authors, they submitted a letter stating that the images in Figure 5 are incorrect and that the current findings require validation through further experiments. All authors agreed with this and signed the retraction letter. However, our investigation uncovered multiple instances of image duplication with unrelated papers from different institutions. Specifically:

• The transwell assay images in Figure 5A, 5B were also found in previously published papers [1–4].

• The immunofluorescent images in Figure 5B are the duplicates of images in Figure 5K of a 2016 paper [5] and in Figure 3D of a concurrently published paper [6].

• The Flow Cytometry images in Figure 5C were also found in earlier published papers [4, 7, 8], two of which are already retracted.

• The images of the wound healing assay in Figure 6C were present in unrelated and already retracted papers [9–11].

• Figures 7A, 7C, presenting western blot data on protein expression in non-small cell lung cancer cells, showed unexpected similarities to western blot bands in Figures 1 and 3–5 of a paper [12] studying lipid accumulation in hepatic cells.

In light of these findings, the editorial decision has been made to retract this paper.
